# Exploring the Effect of the Polyol Structure and the Incorporation of Lignin on the Properties of Bio-Based Polyurethane

**DOI:** 10.3390/polym17050604

**Published:** 2025-02-24

**Authors:** Bomin Kim, Jihoon Lee, Sunjin Jang, Jaehyeon Park, Jinsil Choi, Seungyeol Lee, Joonhoo Jung, Jaehyung Park

**Affiliations:** 1 Interior & Exterior Materials Development Team, Hyundai Motor Group, Hwaseong-si 18280, Republic of Korea; bomin812@gmail.com; 2 Department of Carbon and Fiber Composite Materials, Kyungpook National University, Daegu 41566, Republic of Korea; jsjsjsj0501@gmail.com (S.J.); p0526jh@gmail.com (J.P.); 3 Department of Integrative Biology, Kyungpook Nation University, Daegu 41566, Republic of Korea; ruta5266@gmail.com; 4 Department of Plant Medicine, Kyungpook National University, Daegu 41566, Republic of Korea; chlwlstlf321@gmail.com (J.C.); leesy1123@knu.ac.kr (S.L.); 5 ANPOLY Inc., Pohang 37666, Republic of Korea; jjung@anpolyinc.com

**Keywords:** bio-based polyurethane, lignin, bio-based polyol, waterborne polyurethane dispersion, sustainability

## Abstract

This study developed bio-based waterborne polyurethane (BWPU) dispersions containing lignin as a sustainable filler with castor oil (CO), polycaprolactone diol (PCL), or poly(trimethylene ether) glycol (PO3G). The effects of the polyol structure and the presence of lignin on the mechanical performance, thermal stability, water absorption, ethanol resistance, and UV-blocking capabilities of the resulting BWPU samples were evaluated. The results revealed that lignin affects the molecular packing and interchain interactions of CO-based BWPU, thus improving its tensile strength and thermal stability while reducing its water absorption and ethanol permeability. In the PCL-based BWPU, lignin had a minimal impact on water absorption and ethanol resistance but led to greater UV-blocking ability due to interactions between the semi-crystalline matrix of PCL and the aromatic structure of the lignin. In the PO3G-based BWPU, lignin disrupted the polymer network, increasing its water absorption and reducing its ethanol resistance but significantly improving its elongation and UV-shielding behavior. These results highlight the dual role of lignin as a sustainable reinforcing agent and functional additive in enhancing the properties of BWPU. By tailoring the polyol structure and optimizing lignin use, this study demonstrates a framework for the development of eco-friendly PU composites suitable for use as coatings, barriers, UV-shielding films, and packaging

## 1. Introduction

Polyurethanes (PUs) are a versatile group of polymers containing urethane bonds (-NH-COO-) that are widely used in coatings [1,2,3], adhesives [4], elastomers [5], and foams [6,7]. The material properties of PU, such as its flexibility, mechanical strength, and thermal stability, are strongly influenced by its polyol component, which contains hydroxyl (-OH) functional groups and acts as the soft segment in the polymer matrix [8,9]. However, the use of petroleum-based polyols raises significant environmental and health concerns, encouraging the development of bio-based alternatives such as castor oil (CO), polycaprolactone diol (PCL), and poly(trimethylene ether) glycol (PO3G), which are biodegradable and renewable [10,11,12,13,14]. CO, a natural triglyceride with multiple -OH groups, has a branched molecular structure with a high crosslinking density and mechanical strength, while PCL has an ester–ether combination that increases its thermal stability and mechanical performance, and PO3G has linear ether chains that lead to greater flexibility and elasticity [8,15,16]. These differences in material properties mean that bio-polyols can be selected to fabricate PU tailored for specific applications while addressing sustainability goals.

Waterborne PU (WPU) is an eco-friendly alternative to traditional solvent-based PU that reduces the emission of volatile organic compounds. However, WPU often has weaker mechanical properties, requiring the development of innovative approaches to enhance its performance [2,17]. In this respect, lignin, a naturally abundant polymer derived from plant biomass, has emerged as a promising bio-based filler. The unique aromatic structure and hydroxyl-rich functionalities of lignin allow it to interact with the PU matrix through hydrogen bonding, thus enhancing the mechanical strength, thermal stability, UV resistance, and antimicrobial properties of the resulting PU [18,19,20,21]. Lignin also acts as a gap-filling agent within the polymer matrix, increasing the crosslinking density and reinforcing its structural integrity. As a result, our previous research has demonstrated the benefits of integrating lignin into bio-based PU systems [22]. For example, the combination of CO as the polyol and lignin improves the tensile strength, elongation at break, and thermal stability of the resulting bio-based WPU. However, the effects of changing the chemical structure of the polyol on the properties of lignin-containing WPUs remain underexplored.

This study builds on past findings by systematically investigating the effects of lignin and the bio-polyols CO, PCL, and PO3G on the properties of WPU dispersions. By analyzing the chemical interactions between lignin and polyol structures, this research aims to elucidate the synergistic effects that optimize material performance. The findings thus provide a comprehensive framework for the development of advanced sustainable, high-performance PU composites

## 2. Materials and Methods

### 2.1. Materials

This study utilized a range of bio-based polyols, including castor oil (CO), polycaprolactone diol (PCL), and poly(trimethylene ether) glycol (PO3G), as the bio-polyols for the fabrication of WPU due to their unique chemical structures and suitability for PU synthesis. CO was obtained from Alfa Aesar (Ward Hill, MA, USA). The hydroxyl number of CO is 178.9 mg KOH/g (3.19 mmol/g), as determined by previous research [22]. PCL (Mn = 2000 g/mol) and PO3G (Mn = 2400 g/mol) were sourced from Sigma-Aldrich (Burlington, MA, USA). The hydroxyl number of PCL is reported to be 51.0–63.0 mg KOH/g by the supplier. For PO3G, the supplier does not provide explicit hydroxyl number data; however, based on its molecular weight, the estimated hydroxyl number is 23.38 mg KOH/g. Lignin, in the form of sulfonic acid calcium salt, was also acquired from Sigma-Aldrich (St. Louis, MO, USA). The molecular weights of CO (1625 g/mol) and lignin (2029 g/mol) were determined using gel permeation chromatography (GPC), confirming their suitability for the intended polymerization process [22]. While PCL and PO3G are classified as diols and CO as a triol, they are collectively referred to as polyols throughout this study to maintain clarity in comparing their effects on polyurethane properties. This approach ensures that the discussion remains focused on the structural differences among the polyols and their impact on polymer performance, rather than solely distinguishing them by hydroxyl functionality.

The chemical reagents isophorone diisocyanate (IPDI), N-methyldiethanolamine (MDEA), and diethanolamine (DEA) were procured from Samchun Chemical (Seoul, Republic of Korea). Dibutyltin dilaurate (DBTDL) was obtained from Junsei Chemical (Tokyo, Japan) for use as a catalyst, while the solvents methyl ethyl ketone (MEK) and acetic acid (AA) were supplied by Daejung Chemicals (Busan, Republic of Korea). All materials were used as received without further purification, ensuring consistent and reproducible synthesis conditions throughout the study.

### 2.2. Synthesis of Bio-Based Waterborne Polyurethane (BWPU) Dispersions

Bio-based waterborne polyurethane (BWPU) dispersions were synthesized using a carefully controlled process designed to minimize environmental impact while ensuring reproducibility. IPDI (0.02 mol) and MDEA (0.0089 mol) were first combined in a dry 500 mL three-necked round-bottom flask and homogenized at 50.0 °C under mechanical stirring for 50.0 min. Following this, 0.0049 mol of CO, 0.0074 mol of PCL, or 0.0074 mol of PO3G was added to the reaction mixture, and polymerization was carried out at 65.0 °C for 30.0 min. To facilitate the polymerization process and reduce viscosity, 80.0 µL of DBTDL and 57.0 mL of MEK were added, respectively. DEA (0.0014 mol) was then introduced, and the reaction was maintained under continuous stirring at 65.0 °C for 3.00 h. After cooling to ambient temperature, 0.015 mol of AA was added as a neutralizing agent, followed by dispersion into 100.0 mL of deionized water under a constant stirring speed of 400 rpm for 12.0 h to ensure complete homogenization. This process yielded CO-based, PCL-based, and PO3G-based BWPU dispersions (COWPU-0, PCLWPU-0, and PO3GWPU-0, respectively, where “0” indicates the absence of lignin).

To produce COWPU, PCLWPU, and PO3GWPU dispersions with 5 wt% lignin (COWPU-5, PCLWPU-5, and PO3GWPU-5, respectively), lignin was dispersed in deionized water to 5 wt% of the total solid content during the production of the aqueous dispersions. This level of lignin was selected based on prior studies that demonstrated that it provided the optimal balance of mechanical properties and functional performance (e.g., mechanical strength, thermal stability, and UV resistance) [22]. Residual MEK was removed using a rotary evaporator, yielding an eco-friendly BWPU dispersion with a total solid content of 10 wt%. The molar ratio of the chemical constituents and the synthesis conditions were optimized to ensure consistency across batches (Table 1). This optimization was based on previous research on the factors influencing polymerization efficiency and material performance, such as the reactant ratio and processing conditions [22]. Adopting these parameters allowed for precise control over the material properties of the BWPU dispersions, and for the effects of the polyol type and addition of lignin incorporation to be accurately evaluated.

### 2.3. Characterization

The molecular weights (Mn and Mw) and polydispersity index (PDI) of the synthesized BWPU samples were determined using gel permeation chromatography (GPC) on an Alliance e2695 system (Waters Corporation, Milford, MA, USA). The analysis was performed using tetrahydrofuran as the mobile phase at a flow rate of 1.00 mL/min and a controlled temperature of 35.0 °C. The system was calibrated using a polystyrene-based calibration curve, constructed with a series of monodisperse polystyrene standards (Mp = 1000 to 4,000,000 g/mol, Waters Corp.). The molecular weights were calculated based on the elution profiles of these standards, ensuring accurate molecular weight determination for the BWPU samples. The chemical structures of lignin and the BWPU samples were investigated using Fourier-transform infrared spectroscopy (FT-IR) using a Nicolet 380 FT-IR spectrometer (Thermo Fisher Scientific, Waltham, MA, USA), equipped with a Smart iTR ZnSe crystal for attenuated total reflection (ATR) analysis.

The thermal stability of BWPU was assessed via thermogravimetric analysis (TGA) using a Q500 TGA thermal analyzer (TA Instruments, New Castle, DE, USA) under a nitrogen atmosphere at a heating rate of 10.0 °C/min. The mechanical properties of the BWPU films were evaluated using a Universal Testing Machine (UTM, OTT003, Oriental TM, Ansan, Republic of Korea) with a 20 kgf load cell and an extension rate of 10 mm/min. A minimum of five specimens were tested to ensure statistical reliability. X-ray diffraction (XRD) analysis was performed using a panalytical empyrean diffractometer with CuKα radiation (λ = 1.5418 Å). The X-ray generator was operated at 4 kW, with an X-ray tube voltage of 40 kV and a current of 30 mA. The measurements were conducted over a scan range of 5° < 2θ < 80°, with a step size of 0.02626° and a time per step of 96.39 s. The optical properties were assessed using an OPTIZEN POP UV/visible spectrophotometer (Mecasys Co., Ltd., Daejeon, Republic of Korea) over a wavelength range of 190–800 nm. Water absorption tests were conducted following established protocols. Each BWPU sample was immersed in 100 mL of distilled water for predetermined time intervals (0 h, 20 h, 40 h, 60 h, 80 h, 100 h, and 120 h) until equilibrium swelling was reached. Afterward, the swollen samples were separated and weighed. Water absorption was quantified using the following equation:

$$WA\;\left(\%\right)=\frac{W_{s}-W_{d}}{W_{d}}\times 100$$

where $W_{s}$ is the weight of the sample after immersion in water and $W_{d}$ is its initial dry weight. The ethanol resistance of BWPU films was evaluated by immersing each film sample (10 mm × 20 mm) in 20 mL of absolute ethanol at 25 °C in a sealed Petri dish. The samples were observed at regular intervals (0, 3, 5, and 10 h), and their physical integrity, swelling, and dissolution behaviors were recorded. The ethanol resistance was assessed by measuring the swelling behavior and determining the time until complete dissolution for samples that lost structural integrity.

## 3. Results

Table 2 presents the chemical structure of the polyols utilized in this. CO is characterized by multiple -OH groups and ester functionalities, which are responsible for its reactivity, while PCL is a linear polyol that combines both ester and ether functionalities, thus it exhibits high thermal stability and toughness. In contrast, PO3G has a linear ether-only structure, which leads to PUs with enhanced flexibility and elasticity. These structural differences lead to variation in the reactivity and physicochemical properties of the polyols, significantly influencing the synthesis and performance of the resulting BWPU. This variability is in accordance with prior findings on the impact of the polyol composition on PU properties [8,15,16].

**Scheme 1:**
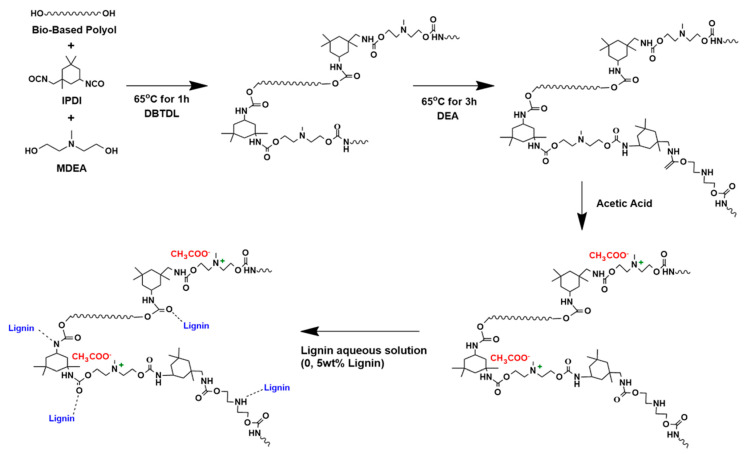
Synthetic process for bio-based waterborne polyurethane (BWPU) dispersions.

Scheme 1 illustrates the process used to synthesize the BWPU samples with lignin. Synthesis begins with the reaction between the NCO groups of IPDI and the -OH groups of MDEA, producing primary urethane bonds. The polyol then reacts with the remaining NCO groups to produce a prepolymer. DEA subsequently extends the chain, inducing secondary urethane bonds and producing the PU backbone. Neutralization with AA and the addition of an aqueous lignin solution result in the final BWPU dispersion. This method differs from traditional PU synthesis, which typically starts with the reaction between a polyol and isocyanate to form a prepolymer.

In the present study, because the three reactive -OH groups of CO significantly increased the viscosity during the chain extension process, an extender was introduced before the polyol, allowing prepolymer formation to occur before chain extension. This not only reduced the amount of solvent required by approximately 50% compared to the conventional method but also improved the sustainability of the synthesis process in line with the demand for eco-friendly PU development [22].

**Figure 1:**
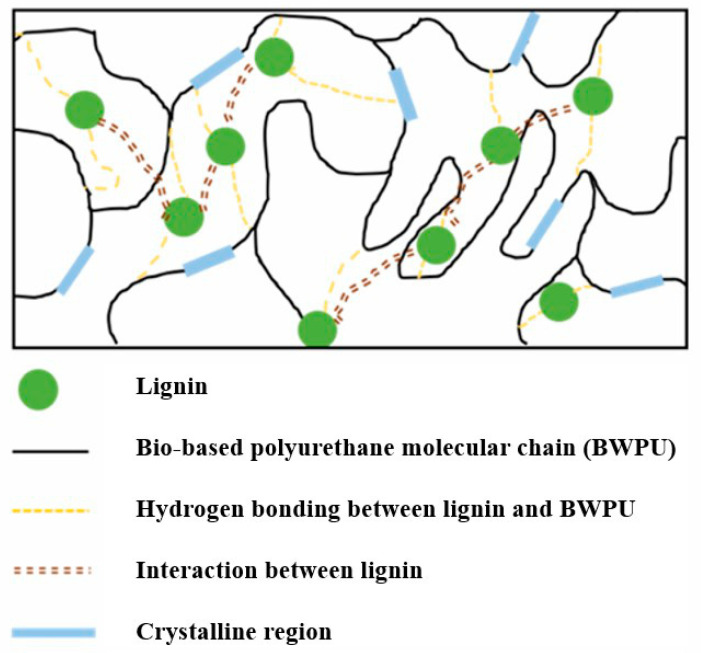
Schematic diagram of the BWPU structure highlighting hydrogen bonding interactions between lignin and the PU matrix, including urethane bonds.

Figure 1 illustrates the morphological transformation of BWPU following the incorporation of lignin. The lignin induces structural changes by infiltrating the amorphous regions of the BWPU and forming hydrogen bonds with the PU molecules, leading to enhanced intermolecular interactions and a more compact polymer network. Previous research has shown that the addition of lignin promotes hydrogen bonding and dynamic interactions within PU matrices, contributing to improved mechanical strength, thermal stability, and UV resistance [18,19,20,21,23]. These results indicate that lignin acts as both a reinforcing agent and an emulsifying stabilizer in BWPU dispersions.

Table 3 summarizes the molecular weights of the synthesized BWPU samples. The polydispersity index (PDI) for these samples ranged from 1.80 to 2.23, representing a relatively narrow molecular weight distribution. However, differences in the number-average molecular weight (Mn) and the weight-average molecular weight (Mw) were observed depending on the polyol used. With CO and PCL, the Mn of the resulting BWPU was approximately 10,000–15,000, while the Mw was 20,000–35,000. In contrast, the PO3G-based BWPU samples had significantly higher molecular weights, with a Mn ranging from 35,000 to 44,000 and a Mw of 65,000 to 100,000.

These results suggest that the structural attributes of the polyols, particularly the branched structure of CO, the linear ester–ether structure of PCL, and the linear ether-only structure of PO3G, significantly influenced the molecular weight during polymerization. The higher molecular weight observed with the use of PO3G can be attributed to its uniform ether structure, which facilitated chain extension and polymerization. The molecular weights obtained via this synthesis method were similar to those reported for conventional WPU synthesis, indicating that the proposed sustainable approach did not negatively affect polymerization efficiency or the resulting properties of the PU [24,25]. Moreover, the incorporation of lignin leads to a notable increase in Mw and Mn, along with a higher PDI in COWPU-5 and PO3GWPU-5, suggesting that lignin promotes increased intermolecular interactions and potential branching in the polymer network. The larger molecular weight distribution observed in these samples suggests that lignin acts as a crosslinking or associating agent, increasing heterogeneity in the molecular weight distribution. However, in PCLWPU-5, a distinct trend is observed, where Mw and Mn decrease upon lignin addition. This deviation may be attributed to lignin interfering with the polymerization process by limiting chain mobility or reducing the efficiency of chain propagation in the PCL-based system. The semi-crystalline nature of PCLWPU might also restrict lignin’s integration into the polymer matrix, resulting in a lower molecular weight compared to the other systems.

**Figure 2:**
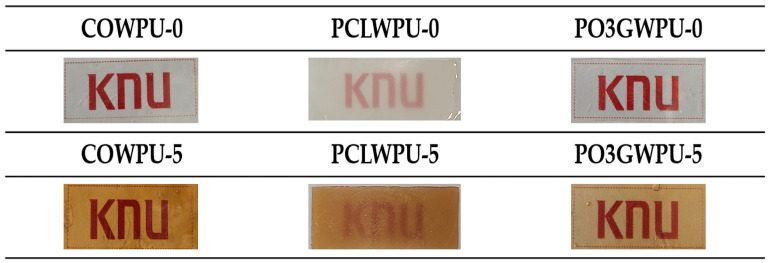
Visual appearance of the prepared BWPU films.

Figure 2 presents images of the prepared BWPU films. Notably, the COWPU-0 and PO3GWPU-0 films had high transparency due to the homogeneity of their polymer matrices. The introduction of lignin to COWPU-5 and PO3GWPU-5 led to a distinct brown coloration due to the natural pigmentation of lignin. This change in color indicated the successful integration of lignin into the PU matrix. In contrast, the PCLWPU films had an opaque white appearance, likely due to phase separation resulting from the semi-crystalline nature of PCL. In PCLWPU-5, the addition of lignin resulted in a dark brown film due to lignin–PU interactions within the semi-crystalline matrix. This variation in appearance between the BWPU films was the result of the interaction between the polyol structure, microstructure, and lignin.

**Figure 3:**
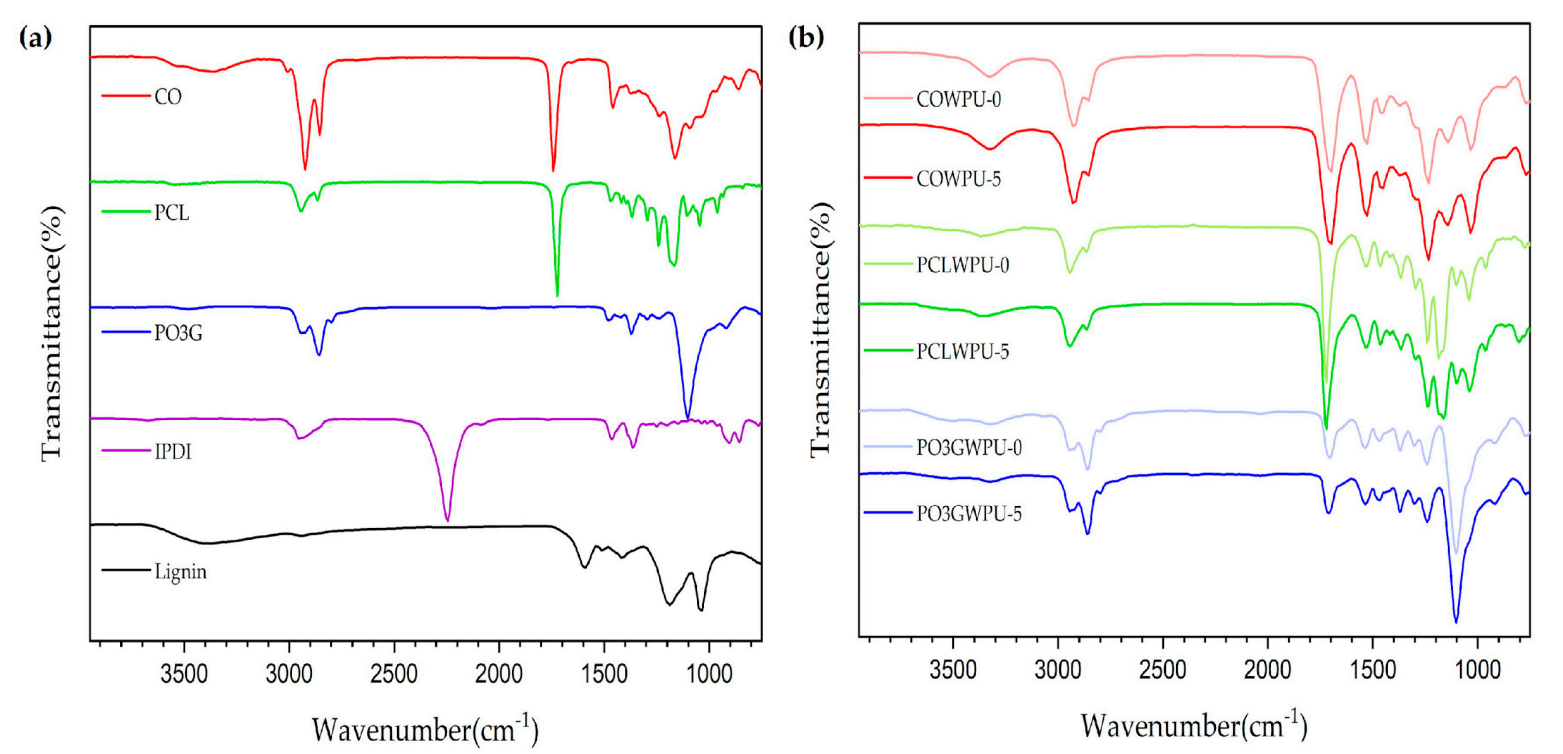
FT-IR spectra for (**a**) the bio-based polyols CO, PCL, PO3G, and lignin, and (**b**) the synthesized BWPU samples.

Figure 3a displays the FT-IR spectra for CO, PCL, PO3G, and lignin, highlighting their characteristic functional groups. The polyols all exhibited prominent CH- and CH2 stretching vibration peaks at 2930 cm^−1^ and 2850 cm^−1^, respectively, as well as a broad -OH stretching peak between 3300 and 3500 cm^−1^. The FT-IR spectra for CO and PCL also contained absorption peaks for ester (C=O-O) bonds in the range of 1720–1740 cm^−1^, while PO3G had a distinct ether (C-O-C) absorption peak at 1103 cm^−1^, which was consistent with its chemical structure [26]. The lignin spectrum contained absorption peaks at 1419, 1511, and 1590 cm^−1^ corresponding to aromatic ring vibrations, while the lignin-specific peaks for the C-O stretching vibrations of the guaiacol ring near 1190 cm^−1^ and C-O stretching near 1050 cm^−1^ and 1182 cm^−1^ confirmed its aromatic and functional diversity [22,27,28]. These results highlight the structural differences between the bio-based polyols and lignin, which contributed to the observed interactions in the resulting BWPU samples.

Figure 3b presents the FT-IR spectra for the synthesized BWPU samples, showing the key chemical transformations that occurred during polymerization. The introduction of IPDI was marked by the appearance of a characteristic isocyanate (NCO) absorption peak at 2280–2300 cm^−1^, which then disappeared in the final BWPU spectra. This disappearance, coupled with the emergence of a strong peak at 1710 cm^−1^ associated with C-O-C bonds, confirmed the successful formation of urethane bonds [29]. The presence of a NH stretching peak at ~3330 cm^−1^ further confirmed urethane bond formation [26]. Additionally, the broad -OH stretching vibration band at 3300–3500 cm^−1^, while reduced in intensity, did not completely disappear. This suggests that a significant fraction of hydroxyl groups participated in the polyurethane reaction, but residual hydroxyl functionalities remained, potentially contributing to secondary hydrogen bonding interactions within the polymer matrix. However, no major changes were observed with lignin addition, suggesting that lignin primarily integrates into the polymer network without significantly altering the overall chemical structure. The limited spectral changes can be attributed to the relatively low lignin content and the steric hindrance of lignin’s bulky aromatic structure, which restricts its reactivity and potential for extensive interaction with the polyurethane matrix [30].

These FT-IR results provide direct evidence for successful PU synthesis, with the polyols and lignin contributing to the formation of the PU matrix. The confirmation of the presence of urethane bonds and the observed interactions between the polyols and lignin were in accordance with the expected chemical transformation, illustrating the consistency of the synthesis method. The absence of unreacted functional groups also confirmed the high efficiency of the reaction.

**Figure 4:**
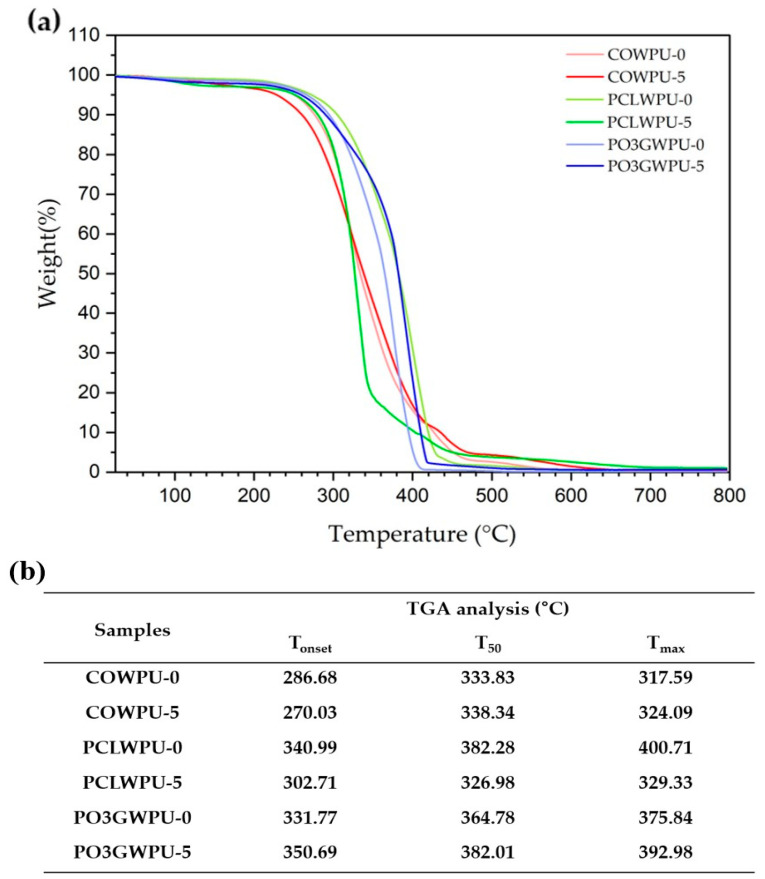
(**a**) Thermogravimetric analysis (TGA) curves and (**b**) a summary of the thermal properties of the BWPU samples.

Figure 4a displays the thermal characteristics of the BWPU samples, classified based on the employed polyol type, highlighting key thermal stability parameters, including T_onset_, T_50_, and T_max_. Variation in thermal stability can be directly associated with the chemical structure of the polyols and their interactions within the PU matrix. Urethane bond dissociation, which typically occurs between 200 and 300 °C, results in decomposition products such as carbon dioxide, isocyanate, alcohol, and primary and secondary amines [31,32]. The thermal degradation of polyols and lignin is primarily observed within the 300–400 °C range [33]. For the COWPU samples, T_onset_ ranged between 270 and 290 °C, representing moderate thermal stability. For the PCLWPU samples, T_onset_ increased to 300–350 °C due to the linear ester–ether structure of PCL, which enhanced the thermal stability. The PO3GWPU samples exhibited the highest T_onset_ at 330–350 °C, which was attributed to the linear ether structure of PO3G as it promoted greater crosslinking and chain mobility.

The addition of lignin altered the thermal stability of the BWPU samples. For COWPU-5 and PO3GWPU-5, lignin increased T_50_, indicating improved thermal resistance and a slower rate of weight loss during decomposition. This enhancement was likely due to the higher crosslinking density in COWPU-5 and the filler properties of lignin in PO3GWPU-5, which enhanced the thermal barrier effect [34,35]. Conversely, PCLWPU-5 exhibited a decrease in T_onset_, T_50_, and T_max_ with the addition of lignin, indicating lower thermal stability. This was attributed to the transition from ordered to disordered soft segment structures caused by the addition of lignin, which disrupted the crystalline regions and reduced the thermal resistance [36].

**Figure 5:**
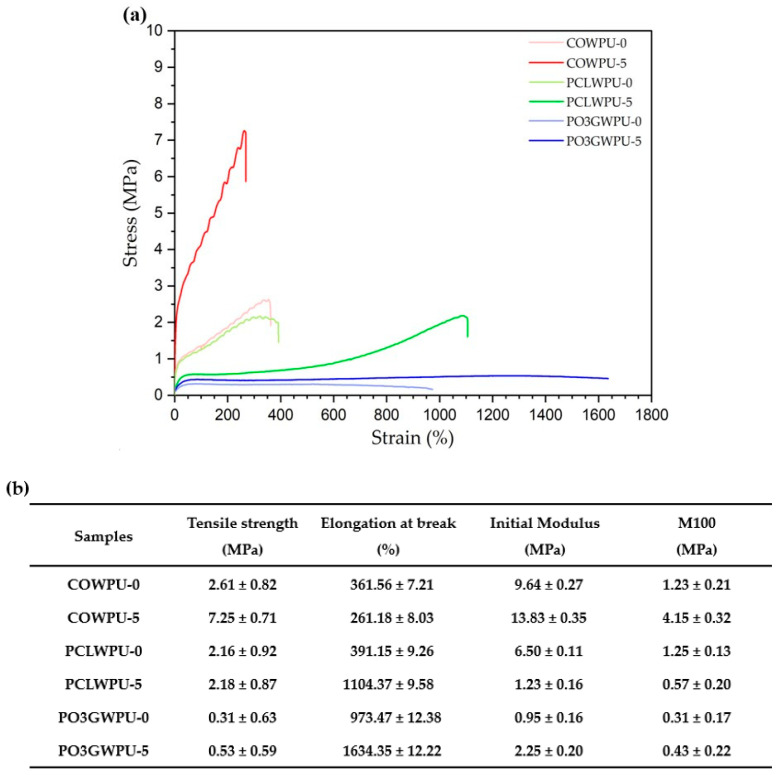
(**a**) Stress–strain curves and (**b**) a summary of the mechanical properties of BWPU films.

**Figure 6:**
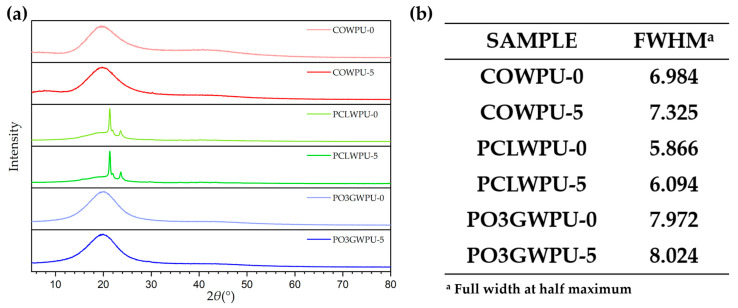
X-ray diffraction (XRD) analysis: (**a**) diffraction profiles and (**b**) FWHM of the BWPU samples.

**Figure 7:**
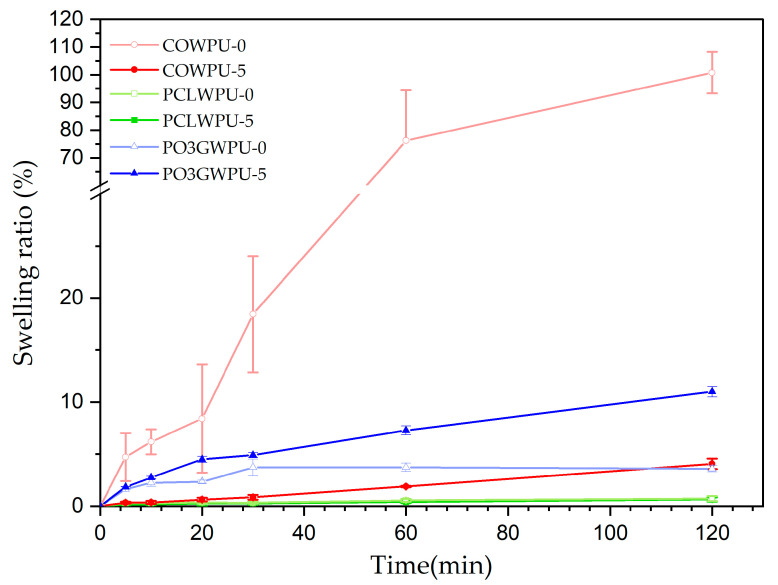
Time-dependent swelling behavior of the BWPU samples.

The mechanical properties of BWPU-X films were evaluated in terms of tensile strength, elongation at breaking point, and modulus (initial and M100), as Figure 5 summarizes. These properties reflect the influence of polyol structure, microstructural organization, and lignin incorporation on the material’s mechanical performance. Among the BWPU-X samples, PCLWPU-0 exhibits a relatively high tensile strength (2.16 MPa) and initial modulus (6.50 MPa), with a M100 value of 1.25 MPa due to its highly ordered microstructure, as confirmed by the XRD results (Figure 6). The distinct diffraction peaks at 2θ = 21°, 22°, and 24° indicate the presence of crystalline regions, which contributes to increased stiffness but reduced elongation at breaking point (391.15%). This behavior aligns with previous studies demonstrating that high crystallinity leads to increased rigidity but lower flexibility in segmented polyurethanes. Conversely, PO3GWPU-0 exhibits a significantly lower tensile strength (0.31 MPa) and an initial modulus (0.95 MPa), with a M100 value of 0.31 MPa but the highest elongation at breaking point (973.47%). The broad and diffuse XRD peaks indicate that PO3G-based BWPU has a highly amorphous structure with minimal crystalline ordering. This amorphous nature allows for greater molecular mobility, leading to enhanced flexibility but lower overall strength. Upon lignin incorporation, mechanical properties exhibit distinct trends depending on the polyol type. In COWPU-X, tensile strength significantly increases from 2.61 MPa to 7.25 MPa, while elongation at break decreases from 361.56% to 261.18%. The increase in tensile strength is attributed to lignin acting as a reinforcing agent, enhancing interchain interactions by hydrogen bonding within the polymer matrix [37,38]. XRD (Figure 6) and swelling behavior (Figure 7) results suggest that lignin incorporation enhances interchain interactions, leading to a more compact polymer matrix, contributing to a higher initial modulus (from 9.64 MPa to 13.83 MPa) and M100 increasing from 1.23 MPa to 4.15 MPa [39].

In contrast, in PO3GWPU-X, lignin addition increases elongation at the breaking point from 973.47% to 1634.35%, while tensile strength shows only a marginal increase from 0.31 MPa to 0.53 MPa. The minimal effect on tensile strength suggests that lignin does not act as a reinforcing filler in this highly amorphous system. Instead, increased molecular disorder leading to reduced packing efficiency, allowing for greater elongation at the breaking point. The initial modulus also increases slightly from 0.95 MPa to 2.25 MPa, while M100 increases from 0.31 MPa to 0.43 MPa, likely due to the formation of additional hydrogen bonds between lignin and the polymer matrix. A unique trend is observed in PCLWPU-X, where lignin incorporation does not significantly change tensile strength (2.16 MPa to 2.18 MPa), but elongation at breaking point increases drastically from 391.15% to 1104.37%, while the initial modulus decreases from 6.50 MPa to 1.23 MPa. This behavior suggests that lignin disrupts crystalline ordering while maintaining sufficient intermolecular interactions, leading to enhanced flexibility without severely compromising structural integrity.

A unique trend is observed in PCLWPU-X, where lignin incorporation does not significantly change the tensile strength (2.16 MPa to 2.18 MPa), but the elongation at breaking point increases drastically from 391.15% to 1104.37%, while the initial modulus decreases from 6.50 MPa to 1.23 MPa. The increase in elongation and decrease in initial modulus suggest that lignin disrupts the semi-crystalline ordering of PCL domains, increasing chain mobility while maintaining sufficient intermolecular interactions to prevent significant mechanical degradation. This disruption is supported by XRD analysis (Figure 6), where broadening of the diffraction peaks is observed, indicating a reduction in ordered crystalline regions.

The differences in the mechanical behavior observed for the COWPU, PCLWPU, and PO3GWPU samples were attributed to variation in the polyol structure and its interaction with lignin. The three -OH groups in CO create a PU elastomer network, enhancing rigidity and tensile strength when lignin is added. Conversely, the two -OH groups in PCL form a linear PU elastomer, which allows for more chain mobility and flexibility, while the uniform ether structure of PO3G promotes elongation due to reduced steric hindrance and enhanced segmental motion [16]. These structural differences demonstrate the dynamic effects of lignin on PU matrices. The incorporation of lignin into these matrices induces complex effects that vary depending on the polymer structure, influencing the balance between molecular interactions and segmental mobility. The observed mechanical trends confirm that lignin does not universally act as a reinforcing agent but rather modulates polymer network organization, either increasing densification (COWPU-X), disrupting crystalline order (PCLWPU-X), or increasing flexibility (PO3GWPU-X). These structural differences demonstrate the dynamic effects of lignin on PU matrices.

XRD analysis was performed to evaluate the structural organization of BWPU-X samples and to understand the impact of lignin incorporation. Figure 6a shows the XRD spectra, while Figure 6b presents the full width at half maximum (FWHM) values to quantify structural variations. COWPU-0 and COWPU-5 exhibit broad amorphous peaks at 2θ = 18–19°, indicating a largely disordered structure [22,40,41]. The FWHM values increased from 6.984 to 7.325 upon lignin incorporation, suggesting a decrease in the average crystalline domain size, or that enhanced hydrogen bonding and π-π stacking interactions compensate for the loss of ordered domains, resulting in a denser polymer network. This structural change contributes to enhanced intermolecular interactions, reinforcing the polymer matrix and increasing modulus. PCLWPU-0 displays distinct crystalline peaks at 2θ = 21°, 22°, and 24°, confirming its semi-crystalline nature [42]. Upon lignin addition, these peaks remain prominent, but the increased FWHM suggests partial disruption of ordered regions, correlating with increased elongation at break and reduced modulus (Figure 5) [36]. This disruption of the crystalline regions likely leads to greater chain mobility, resulting in increased elongation. PO3GWPU-X samples exhibit broad, diffuse peaks, characteristic of amorphous polymers. The FWHM increase in PO3GWPU-5 further indicates increased molecular disorder and decreased intermolecular packing, which aligns with its higher elongation and swelling ratio (Figure 7). This behavior suggests that lignin incorporation affects the packing efficiency and molecular mobility within the PO3G-based matrix. A comparative analysis of FWHM trends suggests that in COWPU-X, lignin acts as a reinforcing filler by promoting intermolecular interactions and increasing polymer density, whereas in PO3GWPU-X, lignin disrupts the amorphous structure which increases chain mobility. In PCLWPU-X, the semi-crystalline nature restricts the extent of lignin-induced structural modifications, leading to a different mechanical response.

The water absorption of BWPU-X films is a critical parameter that reflects the microstructural organization, polymer network density, and the hydrophilic–hydrophobic balance of the materials. Figure 7 illustrates the swelling ratio of BWPU-X films over time, revealing distinct variations depending on the polyol type and lignin incorporation. COWPU-0 exhibits the highest swelling ratio (~100.77% after 2 h), highlighting its highly amorphous structure and hydrophilic nature. Castor oil contains multiple hydroxyl (-OH) groups, which contribute to enhanced hydrogen bonding with water molecules, increasing water uptake. Additionally, its branched triglyceride structure leaves significant free volume within the polymer matrix. This structural characteristic facilitates extensive water diffusion into the material, leading to high swelling. However, upon lignin incorporation, COWPU-5 shows a dramatic reduction in water absorption (~4.06%), indicating that lignin plays a crucial role in restricting water penetration. The significant decrease in swelling is attributed to lignin infiltrating the amorphous regions of the COWPU matrix and establishing additional hydrogen bonds with polyurethane chains, which enhances crosslinking density and reduces free volume. This densification effect aligns well with the observed increase in the modulus (Figure 5). PCLWPU-0 and PCLWPU-5 exhibit minimal swelling (0.72% and 0.64%, respectively), which can be explained by the high crystallinity of the PCL-based polyurethane network. XRD results (Figure 6) confirm that PCLWPU samples retain their well-defined crystalline peaks, even after lignin incorporation, which restricts water diffusion. The ester–ether structure of PCL contributes to the formation of a semi-crystalline phase, where the ordered crystalline domains act as a barrier to water absorption. Consequently, the swelling ratios of PCL-based BWPUs remain nearly unchanged, demonstrating that the polyol structure plays a dominant role in water uptake behavior in these systems. PO3GWPU-0, in contrast, shows moderate swelling (~3.60%) due to its amorphous nature and the flexible ether linkages in PO3G [8,43].

The absence of crystalline regions allows water molecules to diffuse through the polymer matrix more easily than in PCLWPU-X. Interestingly, PO3GWPU-5 exhibits a further increase in water absorption (~10.99%), suggesting that lignin incorporation reduced molecular packing efficiency within the polymer network rather than reinforcing intermolecular interactions. This results in increased porosity and facilitates greater water uptake. The observed increase in swelling correlates with mechanical test results (Figure 5), where PO3GWPU-5 showed a significant increase in elongation at breaking point, further supporting the hypothesis that lignin acts as a plasticizing agent in this highly amorphous system rather than a reinforcing filler.

These findings indicate that the water absorption behavior of the BWPUs was affected by the complex interaction between lignin and the polyol structure. Understanding these interactions provides valuable insights for the optimization of BWPU formulations for specific applications, such as moisture-resistant coatings that require low water absorption or membranes designed for controlled water permeability.

**Figure 8:**
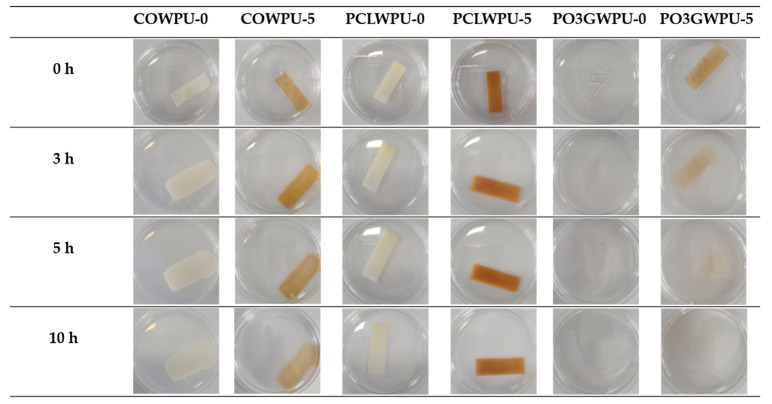
Ethanol resistance of the BWPU samples.

Figure 8 summarizes the ethanol resistance of the BWPU films, revealing significant variation based on the polyol type and the presence or absence of lignin. PO3GWPU had the lowest ethanol resistance, with complete dissolution observed after 10 h. This could be attributed to the dominance of the ether groups in PO3G, which are more susceptible to solvents compared to ester bonds [44]. PCLWPU, which contained both ester and ether groups, demonstrated improved ethanol resistance due to the superior stability of ester bonds in ethanol. In contrast, COWPU, despite containing ester groups, exhibited lower ethanol resistance than PCLWPU, with complete dissolution occurring after 10 h. This lower resistance was attributed to the branched structure of CO, which introduces voids between PU elastomer chains and allows ethanol to penetrate the matrix, weakening its structural integrity [43]. However, the introduction of lignin significantly enhanced the ethanol resistance of COWPU. Comparative images at 3 h and 5 h intervals reveal that swelling was substantially lower for COWPU-5 than for COWPU-0, which was in accordance with the water absorption tests. The addition of lignin enhances the interchain interactions by infiltrating the interface between urethane chains, forming hydrogen bonds and promoting other dynamic interactions. These interactions increase the cohesive strength of the polymer matrix, thus overcoming the affinity between ethanol molecules and urethane chains. Consequently, the structural coherence of the COWPU-5 films was preserved for a longer duration under ethanol exposure.

The ethanol resistance results for the BWPU films demonstrate that the polyol structure and lignin determine their solvent stability. While PO3GWPU and COWPU inherently lacked resistance to ethanol due to their structural vulnerabilities, the introduction of lignin effectively mitigated these weaknesses in COWPU. This highlights lignin’s potential as a reinforcing agent to improve the solvent resistance of BWPUs in applications requiring enhanced durability in solvent-rich environments.

**Figure 9:**
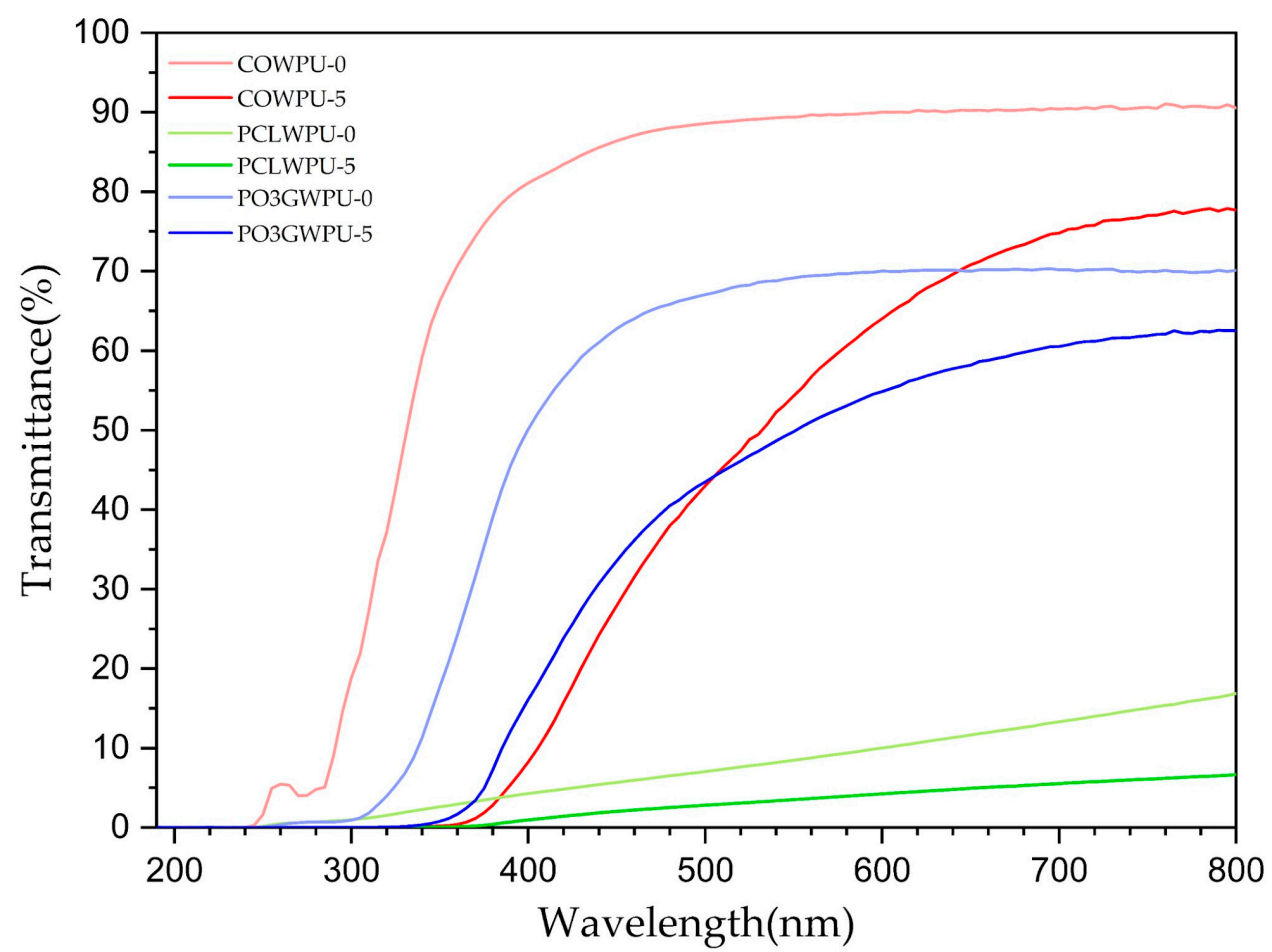
UV transmittance of the BWPU films.

Figure 9 presents the transmittance of the BWPU films, highlighting the influence of a polyol structure and of lignin incorporation on their optical properties. COWPU-0 exhibited a transmittance of approximately 90% at 550 nm, while PO3GWPU-0 reached 70% transmittance at 600 nm, reflecting their amorphous nature and relatively low light-scattering properties. In contrast, PCLWPU-0 exhibited a transmittance below 20% across the visible spectrum, which is attributed to the semi-crystalline nature of PCL, leading to increased light scattering. The addition of lignin to the BWPU samples led to a significant reduction in transmittance for all polyols, as confirmed by the increased opaqueness and darker color profile of the BWPU-5 films compared to their BWPU-0 counterparts (Figure 1). These changes resulted from the aromatic and conjugated structure of lignin, which absorbs and scatters light, reducing transmittance.

The UV spectrum is divided into three regions: UV-A (320–400 nm), UV-B (280–320 nm), and UV-C (100–280 nm). Lignin is enriched in phenolics, quinoid structures, ketones, and intramolecular hydrogen bonds, which offer strong UV-blocking capabilities [45,46]. The BWPU-0 films exhibited limited UV shielding, with minimal attenuation across all UV regions. However, the lignin-containing BWPU-5 films demonstrated significantly enhanced UV-blocking properties regardless of the polyol used. In particular, COWPU-5 and PO3GWPU-5 effectively blocked UV-B and UV-C light, with protection extending up to 360 nm and 340 nm, respectively. Notably, PCLWPU-5 offered the most comprehensive UV protection, blocking UV-A, -B, and -C, with attenuation observed up to 380 nm. The superior performance of PCLWPU-5 could be attributed to the synergy between the semi-crystalline matrix of PCL and the UV-absorbing functionality of lignin.

## 4. Conclusions

This study systematically investigated the effects of the polyol structure and the addition of lignin on the properties of BWPU films, with a focus on their mechanical performance, thermal stability, water absorption, ethanol resistance, and UV-shielding capabilities. The findings highlighted the critical role of polyol chemistry and lignin–PU interactions in determining the functional characteristics of BWPU.

The incorporation of lignin had a variety of effects on the BWPUs depending on the polyol used. In COWPU, lignin significantly enhanced both ethanol resistance and thermal stability by affecting the molecular packing and reducing the free volume within the matrix, leading to lower water absorption and higher structural integrity. In contrast, PCLWPU, with its semi-crystalline structure, exhibited minimal changes in water absorption and ethanol resistance with the addition of lignin but exhibited enhanced UV-blocking properties, which was attributable to the synergistic interactions between the crystalline domain of PCL and the aromatic structure of lignin. PO3GWPU exhibited increased water absorption and reduced ethanol resistance due to the disruption of the polymer network by the -OH groups of lignin, but it also had enhanced elongation and notable UV-blocking capabilities.

This study demonstrates that lignin acts as both a reinforcing agent and a functional additive. Its ability to form hydrogen bonds, infiltrate polymer chains, and enhance interchain interactions contributes to improvements in the mechanical, thermal, and UV-protective properties of PU systems, while also influencing their water and solvent resistance. The findings highlight the interaction between the polyol structure and lignin, providing a robust framework for tailoring BWPU formulations for specific applications.

In conclusion, the incorporation of lignin into bio-based polyol systems offers a sustainable and versatile approach for the development of advanced PU materials with improved performance characteristics. These materials hold significant potential for protective coatings, moisture-resistant barriers, UV-shielding films, and environmentally friendly packaging. Future research should explore the long-term durability and recyclability of these systems to enhance their practicality for use in industrial and commercial applications.

## Figures and Tables

**Table 1:** Composition of the BWPU samples.

Samples	Polyol (Func. mol)	IPDI (Func. mol)	Chain Extender (Func. mol)		Lignin (wt%)	Bio-Based Component (wt%)
			MDEA	DEA		
COWPU-0	1.00	2.75	0.600	0.100	0.00	45.0%
COWPU-5					5.00	47.6%
PCLWPU-0					0.00	72.2%
PCLWPU-5					5.00	73.5%
PO3GWPU-0					0.00	77.1%
PO3GWPU-5					5.00	78.2%

**Table 2:** Chemical structure of the bio-based polyols used in the present study.

Polyols	Chemical Structure
Castor oil (CO)	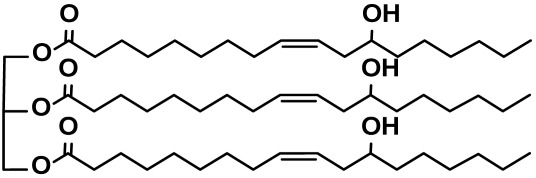
Polycaprolactone diol (PCL)	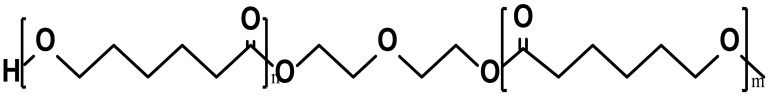
Polyether diol (PO3G)	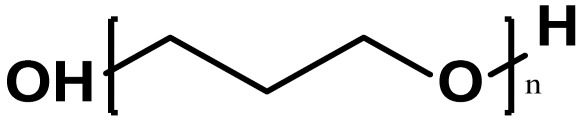

**Table 3:** Gel permeation chromatography (GPC) results for the synthesized BWPU samples.

Samples	Mw	Mn	PDI (Polydispersity Index)
COWPU-0	2.07 × 10^4^	1.10 × 10^4^	1.87
COWPU-5	3.37 × 10^4^	1.60 × 10^4^	2.11
PCLWPU-0	2.37 × 10^4^	1.31 × 10^4^	1.80
PCLWPU-5	2.06 × 10^4^	1.04 × 10^4^	1.98
PO3GWPU-0	6.50 × 10^4^	3.53 × 10^4^	1.84
PO3GWPU-5	9.85 × 10^4^	4.40 × 10^4^	2.23

## Data Availability

The original contributions presented in this study are included in the article. Further inquiries can be directed to the corresponding author.
